# Supplementary material for: Amide Proton Transfer (APT) imaging in tumor with a machine learning approach using partially synthetic data
Source: ArXiv. 2023 Dec 13:arXiv:2311.01683v2. Originally published 2023 Nov 3. Preprint. [Version 2] (PMC10635304)
Supplement: 1 [file NIHPP2311.01683V2-supplement-1.pdf]

Supporting information

Supporting information Theory: Multiple-pool Lorentzian fitted R_{eff} and R_{ex}^{MT}

In a multiple-pool model including CEST/NOE, DS, and MT effects, these effects acquired in steady state can be described simultaneously by superimposing their rotating frame relaxations, assuming concentrations of the CEST/NOE and MT pools are much less than 1 (1,2). The equation is as follows:

$$R_{1\rho}(\Delta\omega) \approx R_{eff}(\Delta\omega) + \frac{R_{ex}^{CEST}(\Delta\omega)}{1+f_m} + R_{ex}^{MT}(\Delta\omega) \tag{1}$$

$R_{1\rho}(\Delta\omega)$ can be approximated as:

$$R_{1\rho}(\Delta\omega) \approx \frac{S_0 R_{1obs}\cos^2\theta}{S(\Delta\omega)} \tag{2}$$

By substituting $R_{1\rho}$ from Eq. (2) into Eq. (1), we obtain:

$$\frac{S(\Delta\omega)}{S_0} \approx \frac{R_{1obs}\cos^2\theta}{R_{eff}+R_{ex}^{CEST}(\Delta\omega)/(1+f_m)+R_{ex}^{MT}(\Delta\omega)} \tag{3}$$

The signal $S(\Delta\omega)$ in Eq. (3) can also be considered as a label signal (S_{lab}). In the multiple-pool Lorentzian fit, the quantity $1-S(\Delta\omega)/S_0$ is first calculated before the fitting. From Eq. (3), this quantity can be further expressed as:

$$1-\frac{S(\Delta\omega)}{S_0} \approx \frac{R_{eff}-R_{1obs}\cos^2\theta+R_{ex}^{CEST}(\Delta\omega)/(1+f_m)+R_{ex}^{MT}(\Delta\omega)}{R_{eff}+R_{ex}^{CEST}(\Delta\omega)/(1+f_m)+R_{ex}^{MT}(\Delta\omega)}$$

$$= \underbrace{\frac{R_{eff}-R_{1obs}\cos^2\theta}{R_{eff}+R_{ex}^{CEST}(\Delta\omega)/(1+f_m)+R_{ex}^{MT}(\Delta\omega)}}_{water\ saturaton} + \underbrace{\frac{R_{ex}^{CEST}(\Delta\omega)/(1+f_m)}{R_{eff}+R_{ex}^{CEST}(\Delta\omega)/(1+f_m)+R_{ex}^{MT}(\Delta\omega)}}_{CEST} + \underbrace{\frac{R_{ex}^{MT}(\Delta\omega)}{R_{eff}+R_{ex}^{CEST}(\Delta\omega)/(1+f_m)+R_{ex}^{MT}(\Delta\omega)}}_{MT} \tag{4}$$

This equation can be separated into three terms, each representing a specific effect. The first term represents the water saturation effect, the second term represents any CEST/NOE effects, and the third term represents the MT effect. Since the MT line shape is broader than the CEST/NOE line shape, the second term is modeled as a Lorentzian function. Similarly, the

third term is also modeled as a Lorentzian function. Each term in Eq. (4) has different line width and offset, allowing them to be isolated through the multiple-pool Lorentzian fit.

1) Multiple-pool Lorentzian fitted R_{eff}

When the multiple-pool Lorentzian fitted water saturation effect (the first item in Eq. (4)) is set to zero, the signal S becomes the reference signal for quantifying the water saturation effect (S_{ref_w}). Eq. (4) can be rewritten as,

$$1 - \frac{S_{ref\_w}(\Delta\omega)}{S_0} = \frac{R_{ex}^{CEST}(\Delta\omega)/(1+f_m)}{R_{eff}+R_{ex}^{CEST}(\Delta\omega)/(1+f_m)+R_{ex}^{MT}(\Delta\omega)} + \frac{R_{ex}^{MT}(\Delta\omega)}{R_{eff}+R_{ex}^{CEST}(\Delta\omega)/(1+f_m)+R_{ex}^{MT}(\Delta\omega)} \qquad (5)$$

S_{ref_w} can be derived from Eq. (5) as:

$$\frac{S_{ref\_w}(\Delta\omega)}{S_0} = \frac{R_{eff}}{R_{eff}+R_{ex}^{CEST}(\Delta\omega)/(1+f_m)+R_{ex}^{MT}(\Delta\omega)} \qquad (6)$$

Thus, R_{eff} can be obtained from the ratio of Eq. (3) and Eq. (6),

$$R_{eff} = \frac{S_{ref\_w}(\Delta\omega)}{S_{lab}(\Delta\omega)} R_{1obs} \cos^2 \theta \qquad (7)$$

2) Multiple-pool Lorentzian fitted R_{ex}^{MT}

Since MT line shape is broader than that of CEST/NOE, most signals on the Z-spectrum used to fit the MT effect are far from water ($\Delta\omega > \omega_1$) and are not at the frequency offsets of CEST/NOE. Therefore, in this case, $R_{ex}^{CEST}(\Delta\omega) = 0$ and $R_{eff} \approx R_{1obs}$. Then, the actual fitted item for MT, denoted as $L_6(\Delta\omega)$, can be derived from Eq. (5),

$$L_6(\Delta\omega) = \frac{R_{ex}^{MT}(\Delta\omega)}{R_{1obs}+R_{ex}^{MT}(\Delta\omega)} \qquad (8)$$

Subsequently, R_{ex}^{MT} can be then obtained from Eq. (8):

$$R_{ex}^{MT}(\Delta\omega) = \frac{L_6(\Delta\omega)R_{eff}}{(1-L_6(\Delta\omega))} \approx \frac{L_6(\Delta\omega)R_{1obs}}{(1-L_6(\Delta\omega))} \qquad (9)$$

Supporting information Table S1. Starting points and boundaries of the amplitude, width, and offset of all pools in the Lorentzian fit. The unit of peak width and offset is ppm.

	Start	Lower	Upper
A_{water}	0.9	0.02	1
W_{water}	1.4	0.1	10
Δ_{water}	0	-1	1
A_{amide}	0.025	0	0.2
W_{amide}	0.5	0.4	3
Δ_{amide}	3.5	3	4
A_{amine}	0.01	0	0.2
W_{amine}	1.5	0.5	5
Δ_{amine}	2	1	3
$A_{NOE(-1.6)}$	0.001	0	0.2
$W_{NOE(-1.6)}$	1	0	1.5
$\Delta_{NOE(-1.6)}$	-1.5	-2	-1
$A_{NOE(-3.5)}$	0.02	0	1
$W_{NOE(-3.5)}$	3	1	5
$\Delta_{NOE(-3.5)}$	-3.5	-4.5	-2.5
$A_{semi\text{-}solid\ MT}$	0.1	0	1
$W_{semi\text{-}solid\ MT}$	25	10	100
$\Delta_{semi\text{-}solid\ MT}$	0	-4	4

Supporting information Table S2. Description of the parameters used.

Parameter	Description
f_m	Concentration of the MT pool
f_s	Concentration of solute pool
k_{sw}	Solute-water exchange rate
L_6	Lorentzian fit for MT pool (Supporting Information Theory)
r_{MT}	Scaling factor for MT pool
r_{amines}	Scaling factor for amine pool
R_{1w}	Water longitudinal relaxation rate
R_{eff}	Effective water relaxation in rotating frame
$R_{ex}(\Delta\omega)$	Exchange dependent CEST effect in rotating frame
$R_{ex}^{APT}(\Delta\omega)$	APT effect in rotating frame
$R_{ex}^{MT}(\Delta\omega)$	MT effect in rotating frame
$R_{ex}^{NOE}(\Delta\omega)$	NOE effect in rotating frame
$R_{ex}^{amines}(\Delta\omega)$	Amine CEST effect in rotating frame
S_0	Control signals
S_{lab}	Label signal
S_{ref}	Reference signal

Supporting information Table S3. List of all sample parameters used to create the partially synthetic CEST data using Eq. (3) and Eq. (5). Since the NOE(-1.6) effect is far from the APT effect, it was not included in creating the synthetic training data.

	water	amide	NOE(-3.5)	amines	MT
f_s (%)	100	0.06:0.02:0.14	0.4:0.3:1.6	-	-
k_{sw} (s^{-1})	-	40:30:160	20	-	-
T_1 (s)	1.6:0.2:2.4	1.5	1.5	-	-
T_2 (ms)	40:20:120	2:1:5	0.5	-	-
Δ (ppm)	0	3.6	-3.3	-	-
r	-	-	-	0.5:0.25:1.5	0.4:0.3:1.6

Supporting information Table S4. List of all sample parameters used to create the tissue-mimicking data using numerical simulation of the Bloch-McConnell equation.

	water	amide	NOE(-3.5)	guanidine	amine	MT
f_s (%)	100	0.05:0.04:0.13	0.2:0.6:1.4	0.01:0.02:0.05	0.15:0.15:0.45	4:4:12
k_{sw} (s^{-1})	-	20:60:140	20	300:200:700	3000:2000:7000	25
T_1 (s)	1.5:0.4:2.3	1.5	1.5	1.5	1.5	1.5
T_2 (ms)	30:40:110	1:1.5:4	0.5	15	15	0.03:0.02:0.07
Δ (ppm)	0	3.6	-3.3	2	3	-2.3

Supporting information Table S5. List of all sample parameters used to create the fully synthetic CEST data for type 1 and type 2 simulations. For type 1 simulations, the f_s of guanidine is fixed at 0.03%.

	water	amide	NOE(-3.5)	guanidine	amine	MT
f_s (%)	100	0.06:0.02:0.14	0.4:0.3:1.6	0.01:0.02:0.05	0.2:0.1:0.6	4:4:16
k_{sw} (s^{-1})	-	40:30:160	20	500	5000	25
T_1 (s)	1.6:0.2:2.4	1.5	1.5	1.5	1.5	1.5
T_2 (ms)	40:20:120	1:2:5	0.5	15	15	0.05
Δ (ppm)	0	3.6	-3.3	2	3	-2.3

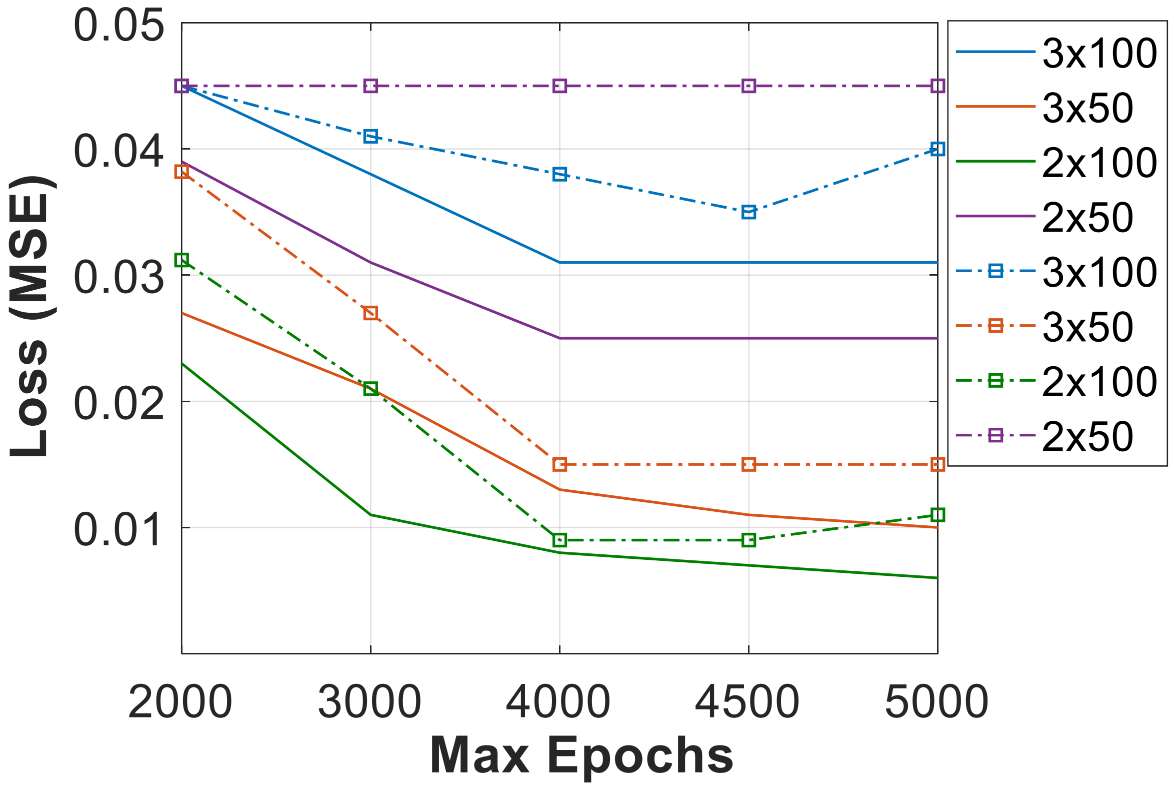

Supporting information Fig. S1 Plot showing the mean squared error (MSE) loss versus the number of epochs for the training and validation using different model configurations. The solid line shows the training loss, while the dashed lines indicate the validation loss. The optimal training was found with 2 layers and 100 neurons with 4000 epochs. The learning rate was kept constant at 1×10^{-3} with Adam optimizer and ReLU activation was used.

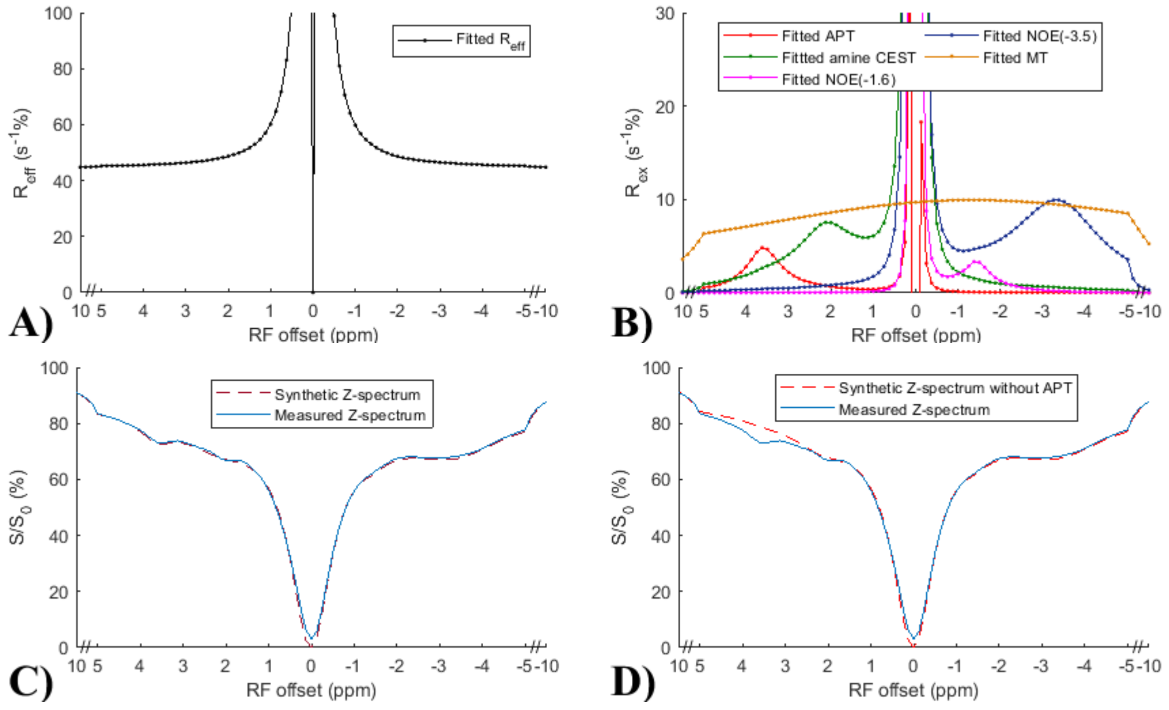

Supporting information Fig. S2 Multiple-pool Lorentzian fitted R_{eff} (a) and fitted R_{ex}^{APT}, R_{ex}^{amines}, $R_{ex}^{NOE(-1.6)}$, $R_{ex}^{NOE(-3.5)}$, R_{ex}^{MT} spectra (b) from the average of the measured CEST Z-spectra in tumors in eight rat brains. Comparison between the measured CEST Z-spectra and synthetic Z-spectrum generated using all multiple-pool Lorentzian fitted components (c) as well as between the measured CEST Z-spectra and synthetic CEST Z-spectrum without APT (d).

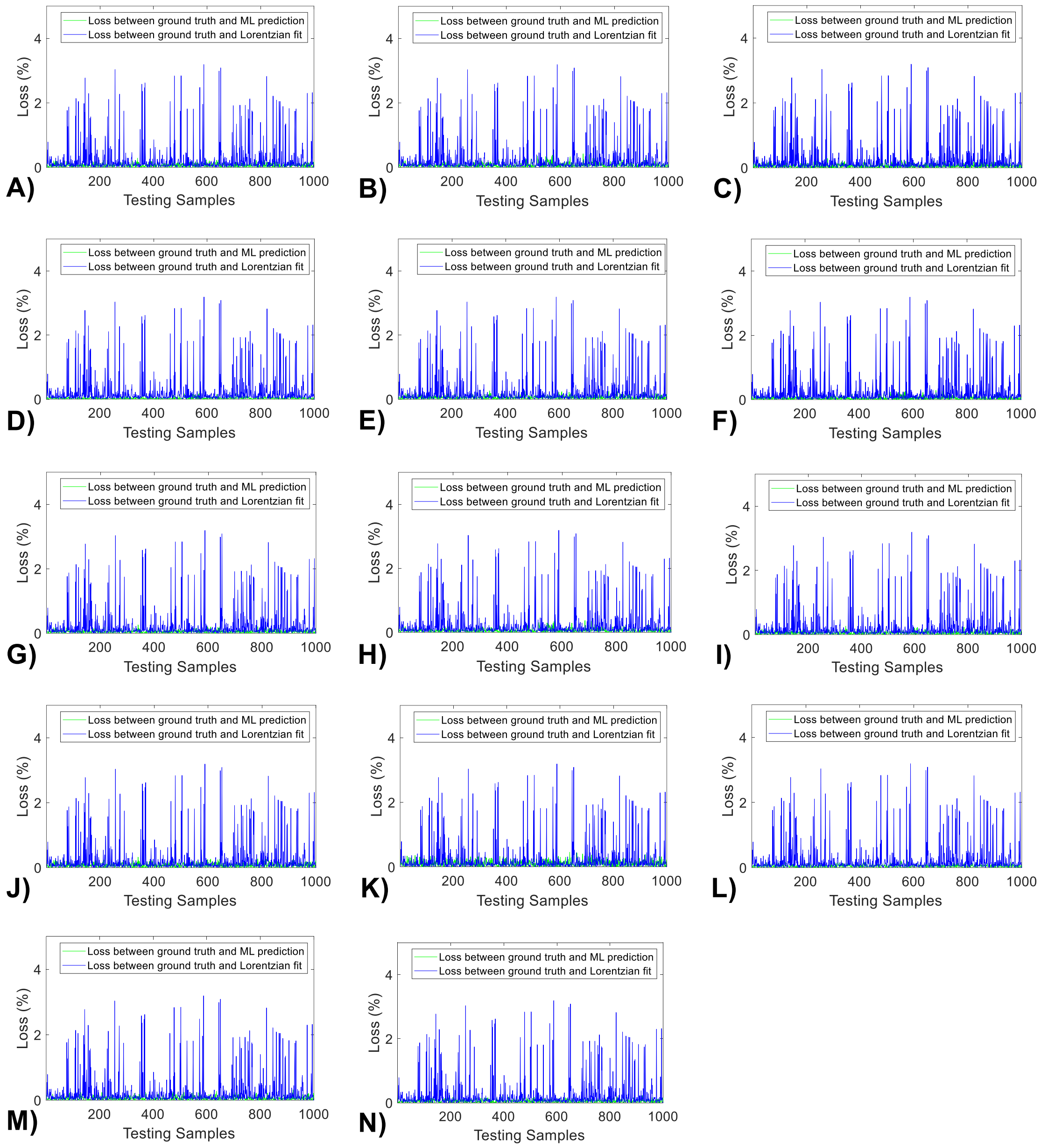

Supporting information Fig. S3 Comparison of losses between the ML method and the multiple-pool Lorentzian fit for all testing data. ML models were trained on fourteen partially synthetic datasets, with the measured components fitted from ten other randomly selected Z-spectra (a-j), as well as the average of 50 (k), 100 (l), 500 (m), and 1000 (n) randomly selected Z-spectra, within the tissue-mimicking data. The mean losses are 7.2148×10^{-4}, 7.2766×10^{-4}, 7.2757×10^{-4}, 7.0909×10^{-4}, and 7.0852×10^{-4} in (a-j), (k), (l), (m), and (n), respectively.

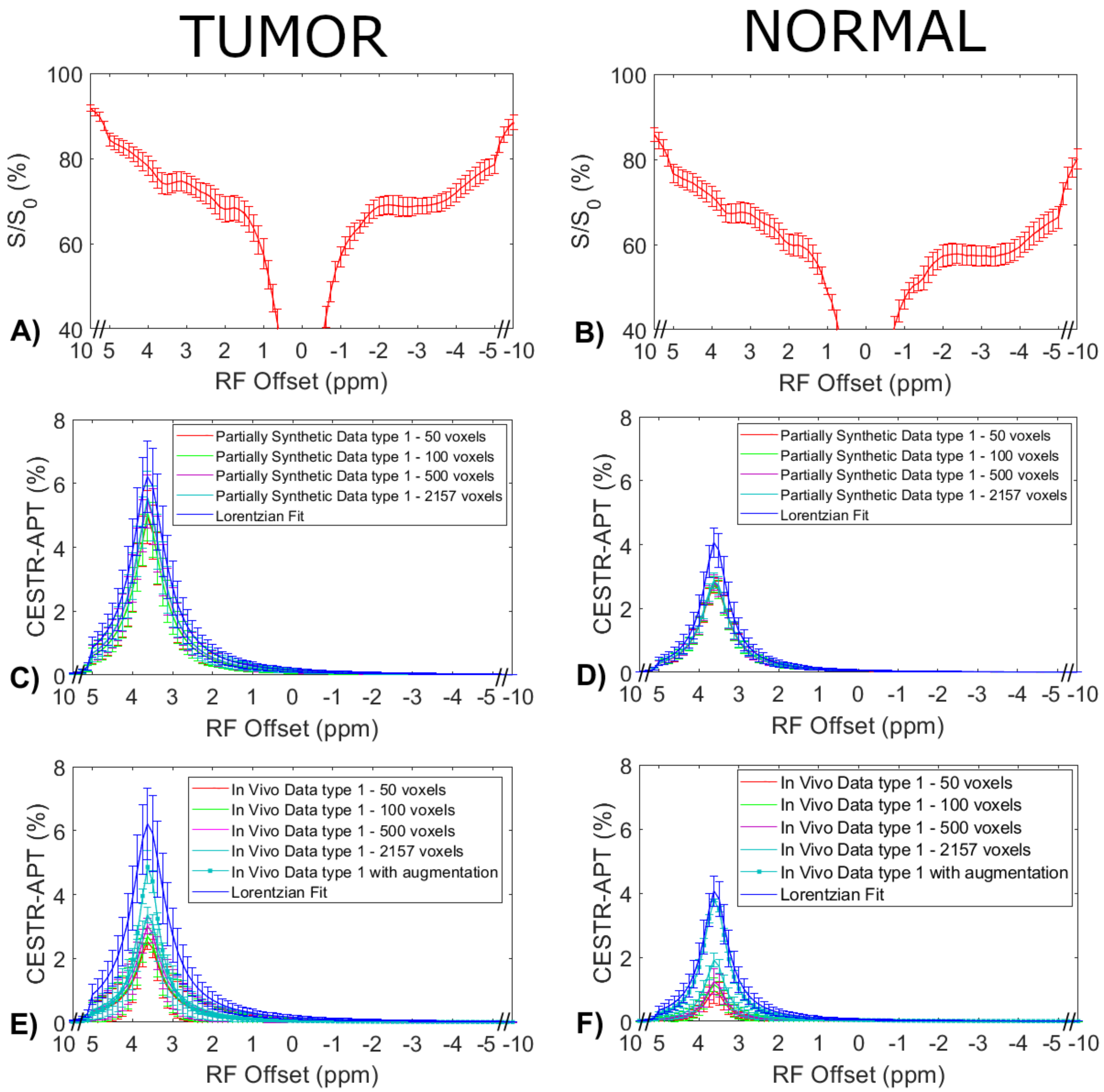

Supporting information Fig. S4 Measured CEST Z-spectra from tumors and contralateral normal tissues in the three testing rats (a, b), the corresponding APT spectra from the ML prediction using the partially synthetic data for type 1 selection of 50, 100, 500, and 2157 voxels within five rat brains (c, d), and the corresponding APT spectra from the ML prediction using the measured in vivo data for type 1 selection of 50, 100, 500, and 2157 voxels within five rat brains as well as with data augmentation (e, f). The multiple-pool Lorentzian fitted APT spectra were also plotted in (c-f) for comparison.

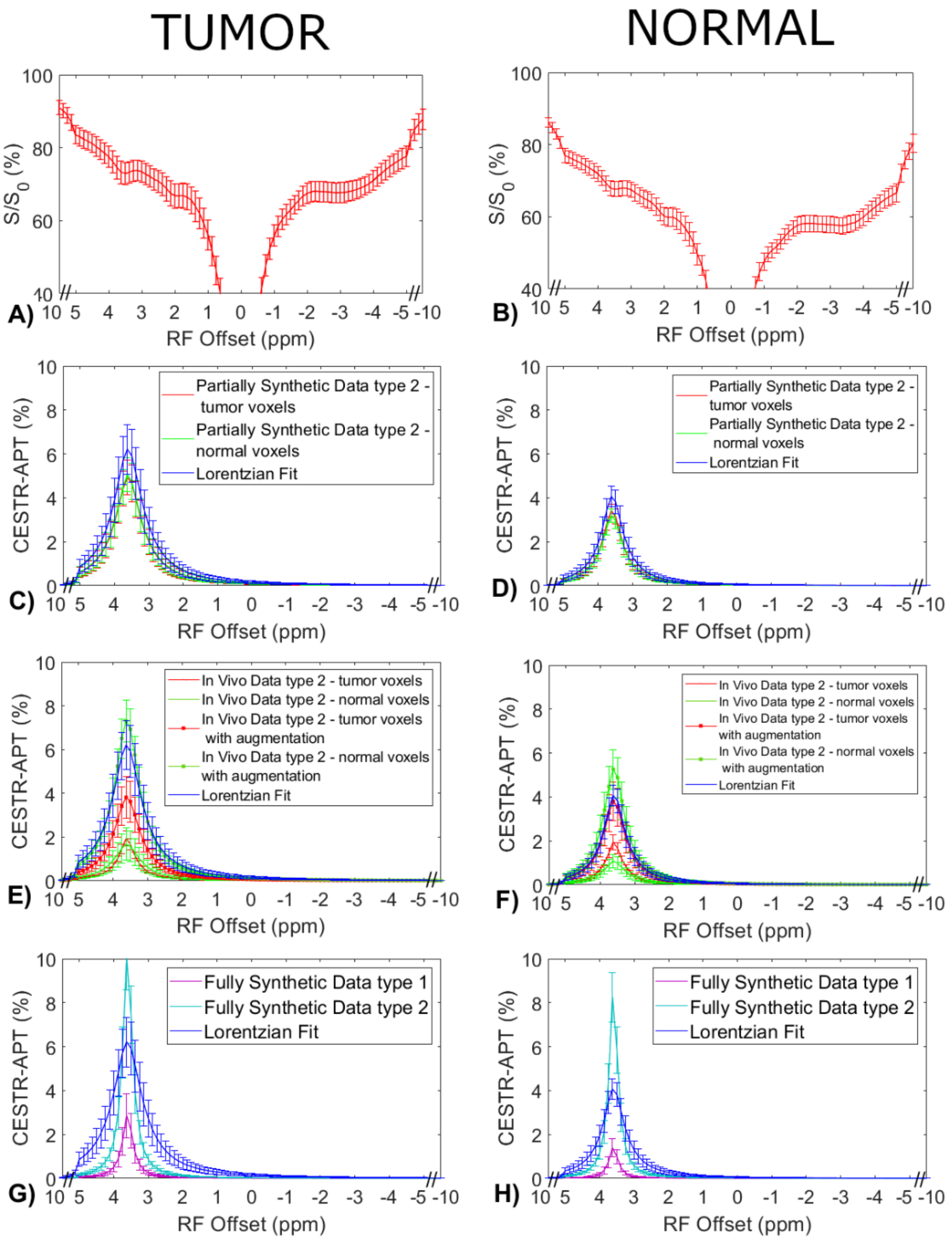

Supporting information Fig. S5 Measured CEST Z-spectra from tumors and contralateral normal tissues in the eight rats (a, b), the corresponding APT spectra from the ML prediction using the partially synthetic data for type 2 selection of tumors and normal tissues (c, d), the corresponding APT spectra from the ML prediction using the measured in vivo data for type 2 selection of tumors and normal tissues (e, f), and ML prediction using fully synthetic data with the type 1 and type 2 simulations (g, h). The multiple-pool Lorentzian fitted APT spectra were also plotted in (c-h) for comparison.

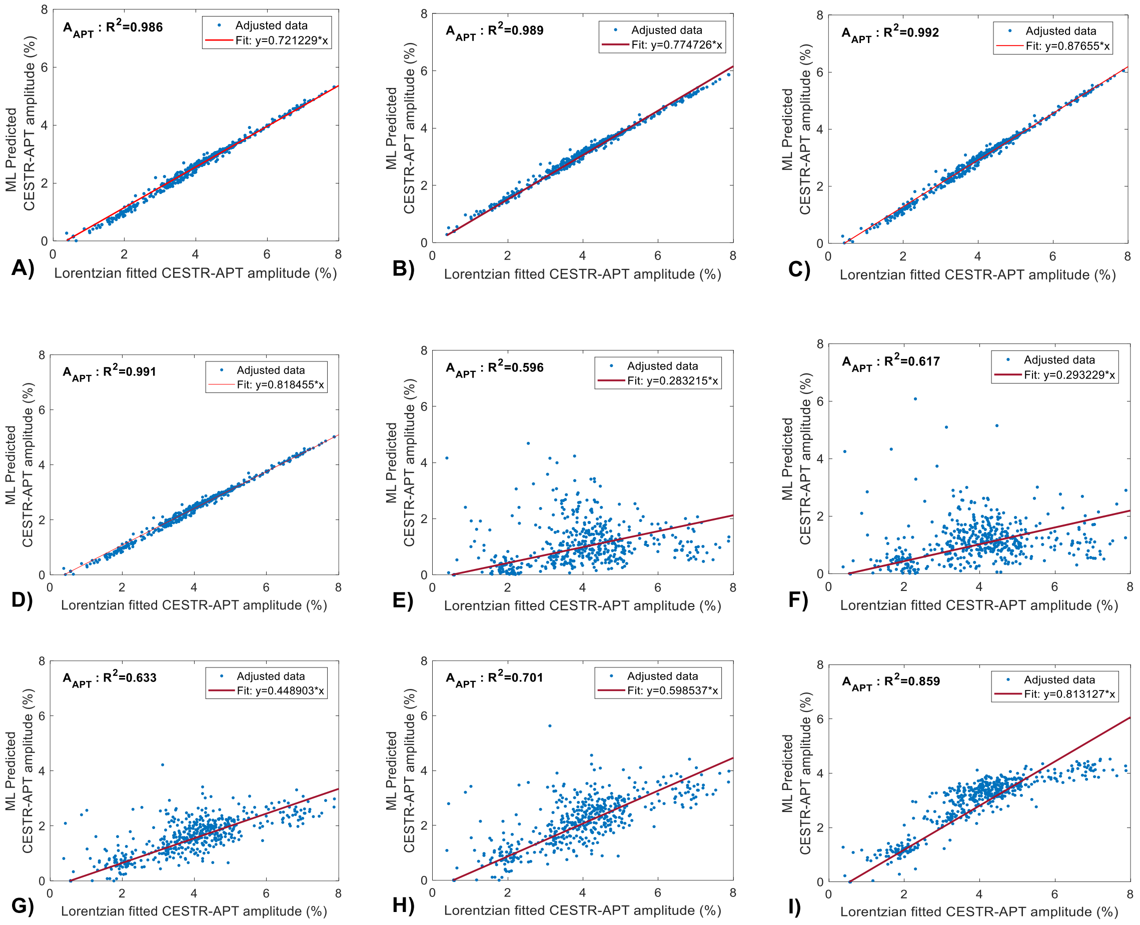

Supporting information Fig. S6 Pixel-wise regression plots between the multiple-pool Lorentzian fitted APT amplitude and the predicted APT amplitude from all the ML models trained using the partially synthetic data for type 1 selection of 50 (a), 100 (b), 500 (c), and 2157 (d) voxels within five rat brains, using the measured in vivo data for type 1 selection of 50 (e), 100 (f), 500 (g), and 2157 (h) voxels within five rat brains as well as with data augmentation (i).

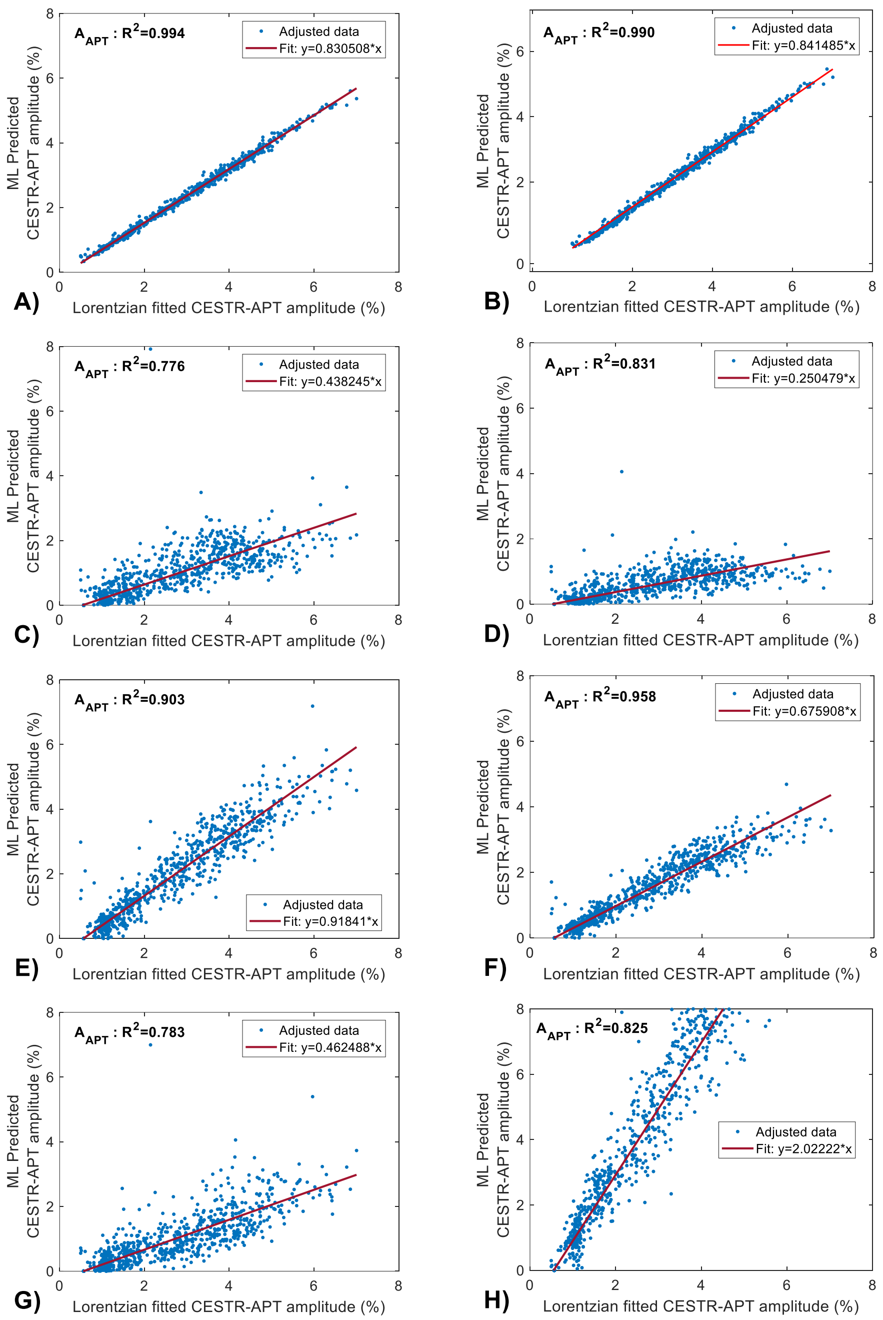

Supporting information Fig. S7 Pixel-wise regression plots between the multiple-pool Lorentzian fitted APT amplitude and the predicted APT amplitude from all the ML models trained using the partially synthetic data for type 2 selection of tumors and normal tissues (a, b), using the measured in vivo data for type 2 selection of tumors and normal tissues (c, d), using the measured in vivo data for type 2 selection of tumors with data augmentation and normal tissues with data augmentation (e, f), and using fully synthetic data with the type 1 and type 2 simulations (g, h).

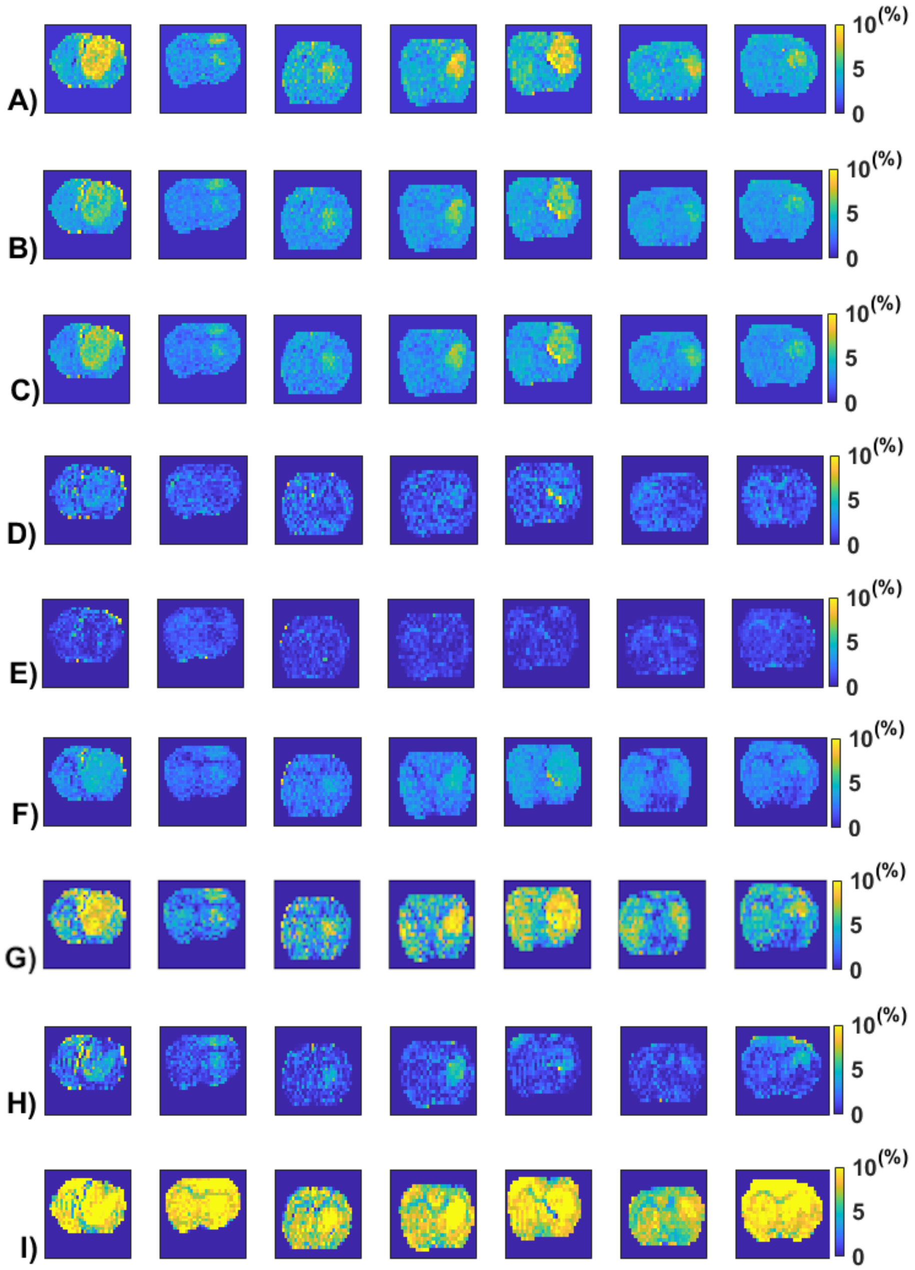

Supporting information Fig. S8 APT amplitude maps from the seven rat brains (from left to right, Rat 2, Rat 3, Rat 4, Rat 5, Rat 6. Rat 7, Rat 8) using the Lorentzian fitting (a), ML prediction using partially synthetic data with the measured components from the in vivo data with type 2 selection of tumors (b) and normal tissues (c), ML prediction using the measured in vivo data with type 2 selection of tumors (d) and normal tissues (e), ML prediction using the measured in vivo data with type 2 selection of tumors with data augmentation (f) and normal tissues with data augmentation (g), as well as ML prediction using fully synthetic data with the type 1 (h) and type 2 (i) simulations.

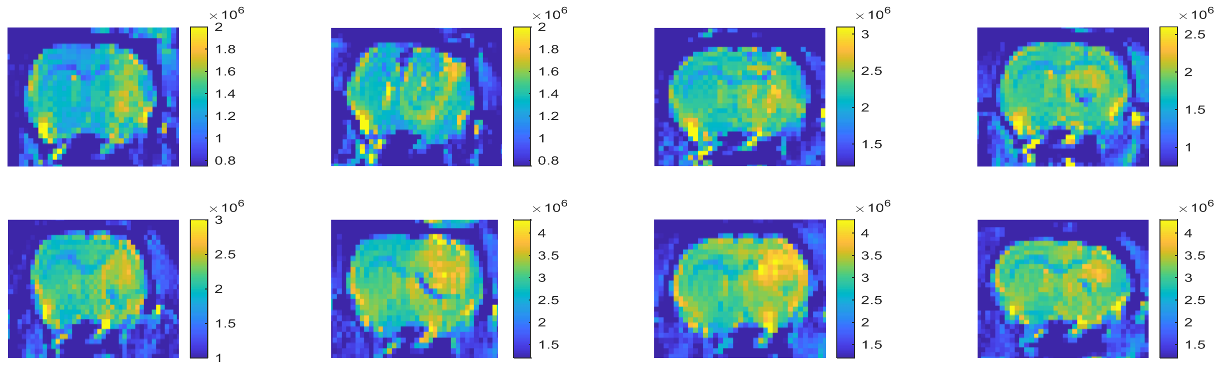

Supporting information Fig. S9 T_2-weighted anatomy images from the eight rat brains. From left to right, Row 1 shows anatomy images for Rat 1 to Rat 4 respectively, and Row 2 shows the anatomy images for Rat 5 to Rat 8 respectively.

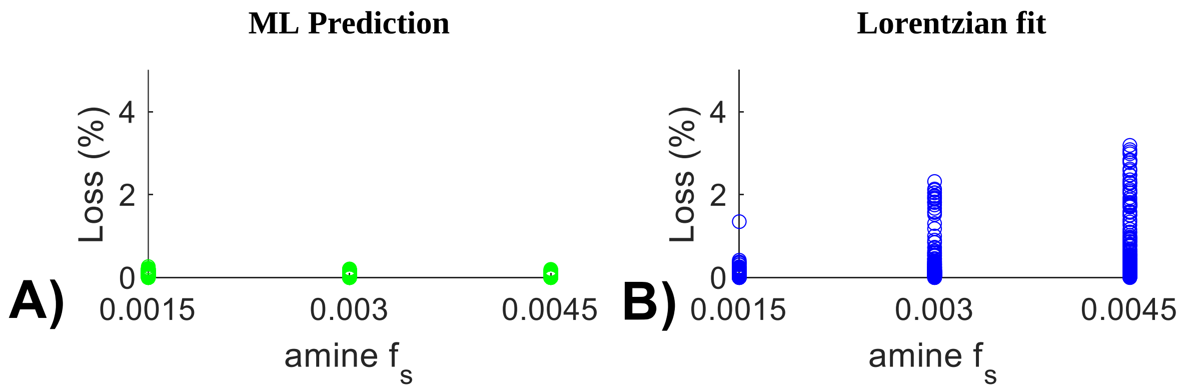

Supporting information Fig. S10 Scatter plots of losses from the ML method using the partially synthetic data (a) and from the multiple-pool Lorentzian fit (b) of all testing tissue-mimicking data vs. amine f_s. The partially synthetic data were obtained with the measured components fitted from a randomly selected Z-spectrum within the tissue-mimicking data.

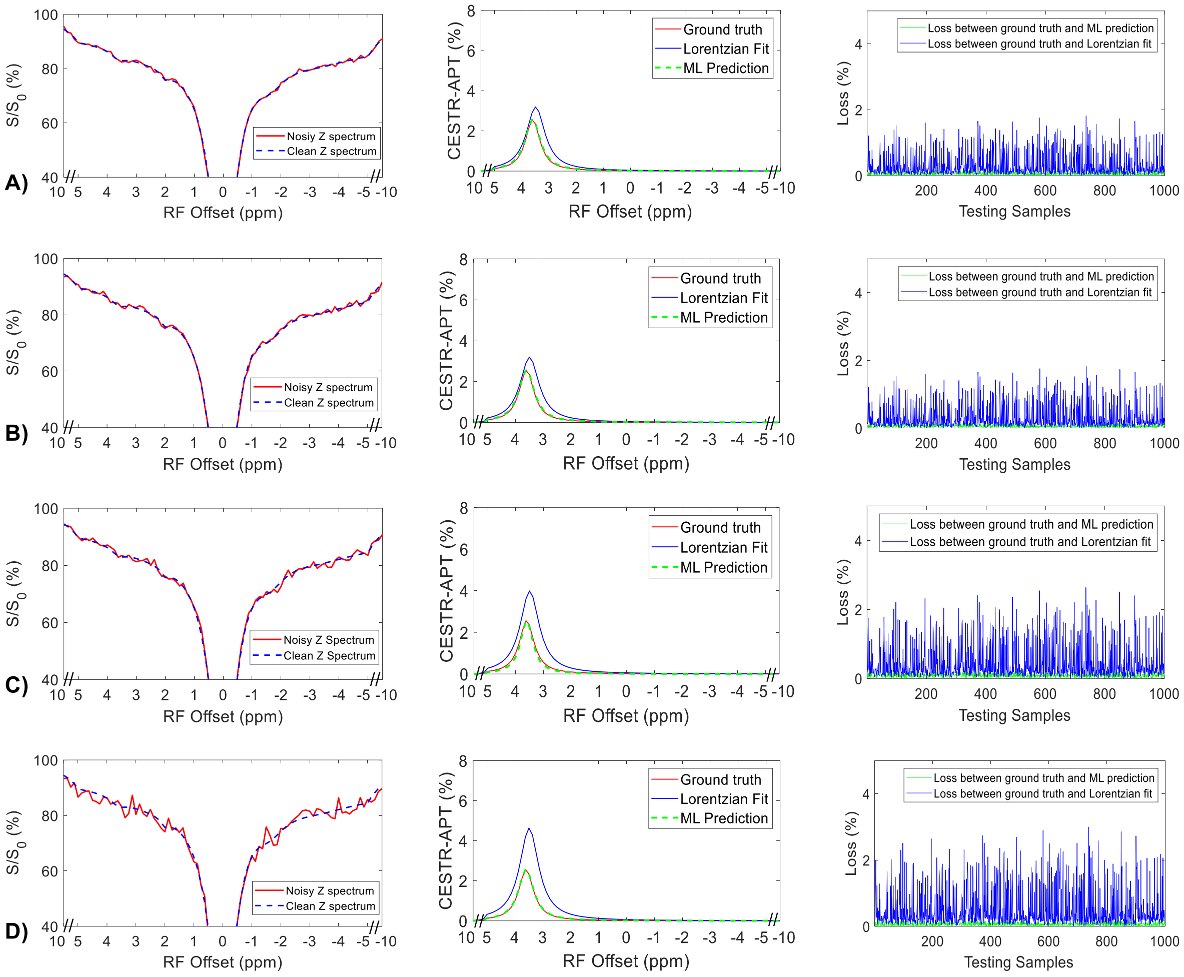

Supporting information Fig. S11 Left column: a representative Z-spectrum from the tissue-mimicking data (clean) together with a few noisy Z-spectra with SNR of 200 (a), 150 (b), 100 (c), 50 (d). Middle column: A comparison of the corresponding APT spectra from the ML prediction, multiple-pool Lorentzian fit, and ground truth. Right column: A comparison of the corresponding losses between the ML prediction and the multiple-pool Lorentzian fit for all testing data.

1. Zaiss M, Zu ZL, Xu JZ, Schuenke P, Gochberg DF, Gore JC, Ladd ME, Bachert P. A combined analytical solution for chemical exchange saturation transfer and semi-solid magnetization transfer. NMR in biomedicine 2015;28(2):217-230.
2. Zaiss M, Bachert P. Exchange-dependent relaxation in the rotating frame for slow and intermediate exchange - modeling off-resonant spin-lock and chemical exchange saturation transfer. NMR in biomedicine 2013;26(5):507-518.
